# A C1 Jefferson Fracture With Vertebral Artery Occlusion and Cerebellar Infarction: A Case Report

**DOI:** 10.7759/cureus.38789

**Published:** 2023-05-09

**Authors:** Mohammed Almalki, Elham A Alghamdi, Reem Alasmari, Norah Aldossary, Turki Hussain, Abdullah Hamad

**Affiliations:** 1 Orthopedic Surgery, King Saud Medical City, Riyadh, SAU; 2 Medicine and Surgery, College of Medicine, Princess Nourah Bint Abdulrahman University, Riyadh, SAU; 3 Medicine, Princess Nourah Bint Abdul Rahman University, Riyadh, SAU

**Keywords:** vertebral artery compression, jefferson fracture, cerebellar infarction, atlas fracture, vertebral artery injury, blunt cervical trauma, cervical trauma

## Abstract

*Jefferson fracture* is a C1 fracture, which happens when an axial load is from the occiput downward to the C1 ring. Usually, it causes outward displacement of the C1 arch, which can injure the vertebral artery. We present a Jefferson fracture with vertebral artery injury, resulting in an asymptomatic ischemic stroke of the left cerebellum. Usually, vertebral artery injuries are asymptomatic since the contralateral vertebral artery and the collateral arteries will adequately supply the cerebellum. Vertebral artery injury (VAI) is typically treated with conservative management with anticoagulants and antiplatelet therapy.

## Introduction

Jefferson fracture is a traumatic axial load from the occiput downward on the arch of the first cervical vertebrae. It causes outward displacement of the C1 ring, frequently accompanied by other upper cervical spine injuries [1]. The incidence of atlas fractures in the cervical spine ranges from 2% to 13% and that of spinal injuries ranges from 1% to 2% [1]. There is an estimated 0.5% chance that a trauma patient will suffer a vertebral artery injury (VAI). Up to 70% of all traumatic VAIs will be associated with a cervical spine fracture, which leads to the spinal cord, brain stem, and brain ischemia [2]. The occlusion of the vertebral artery (VA) seldom causes any symptoms as the opposing VA and the circle of Willis provide adequate collateral blood supply [3]. Although most of these damages are asymptomatic, some might cause a vertebrobasilar infarction, which manifests as dizziness, visual changes, ataxia, or decreased level of consciousness. It has been reported that VA occlusion symptoms typically appear within 24 hours of trauma [3]. Despite the prevalence of other cervical injuries associated with these injuries, atlas fracture treatment remains controversial. As far as we know, no standards or guidelines exist for treating C1 fractures alone or in conjunction with other cervical spine injuries. In C1 vertebrae fractures, surgical intervention is rarely required unless associated with significant upper cervical spine instability [1].

To our knowledge, C1 fracture has been associated with only a few cases of VAI [4]. The authors report a case of cerebellar infarction caused by a fracture of the C1 and discuss conservative management options.

## Case presentation

**Figure 1:**
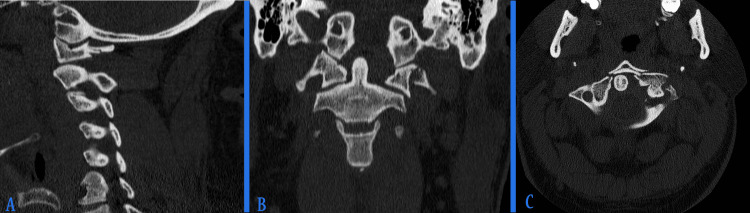
Computed tomography scan shows C1 Jefferson burst fracture extending to the left transverse foramen with atlantoaxial subluxation to the right side: (A) sagittal cut, (B) coronal cut, and (C) axial cut.

A 28-year-old medically free man presented to the emergency department of King Saud Medical City, with a history of pedestrian injury. After advanced trauma life support (ATLS) was applied to the patient and stabilized, a thorough history and detailed physical examination were obtained. He complained of neck and right arm pain. The pain was localized to the neck and right arm without any radiation. No history of head trauma or loss of consciousness was reported. Imaging revealed a C1 Jefferson burst fracture with extension to the left transverse foramen with atlantoaxial subluxation to the right side and a right humeral shaft fracture (Figure 1).

**Figure 2:**
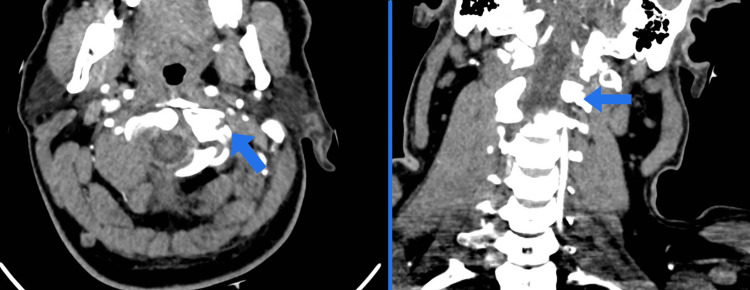
CT neck angiography for the upper cervical spine: the left vertebral artery is close to the fracture with no evidence of extravasation. CT, computed tomography

On examination, the patient was alert and oriented with the Glasgow Coma Scale (GCS) score of 15/15, and the upper and lower limb motor and sensory examinations were intact. Computed tomography (CT) of the neck angiography is part of our protocol for all upper cervical spine fractures (Figure 2).

**Figure 3:**
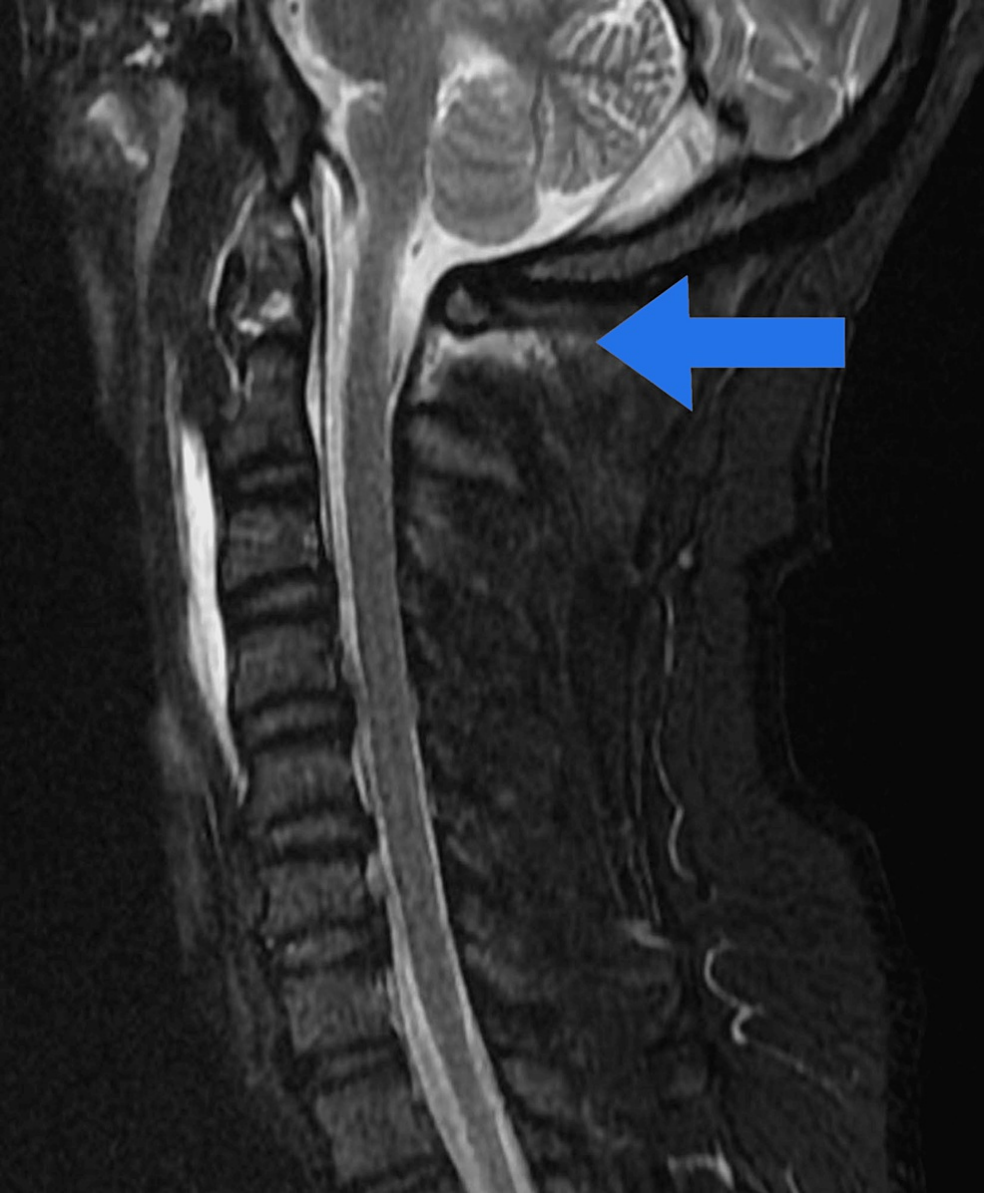
A cervical MRI showing ligamentous injury of the C1 fracture. MRI, magnetic resonance imaging

It revealed that the left vertebral artery is close to the fracture, but no evidence of extravasation suggests arterial injury. A cervical MRI showed ligamentous injury of the C1 fracture, and there was an incidental finding of partial left posterior inferior cerebellar artery (PICA) territory acute infarction (Figure 3).

**Figure 4:**
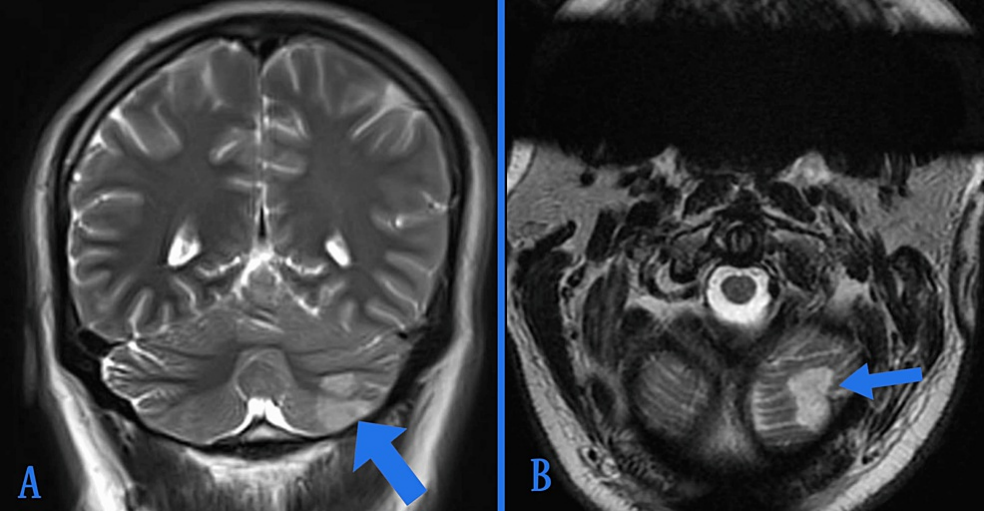
Brain MRI showing a left cerebellar subacute ischemic nonhemorrhagic infarction, pointed with an arrow: (A) coronal cut and (B) axial cut. MRI, magnetic resonance imaging

As a result, the neurology team started the patient on stroke workup and anticoagulant and antiplatelet therapy. Brain MRI showed a left cerebellar subacute ischemic nonhemorrhagic stroke, posing a moderate risk for general anesthesia (Figure 4).

**Figure 5:**
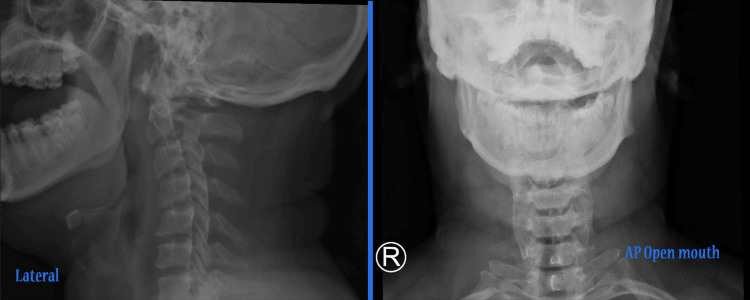
Anterior/posterior open mouth and lateral radiograph follow-up after six weeks of injury.

Operative treatment and conservative treatment were discussed thoroughly, and the patient preferred conservative management. He was placed on a rigid cervical collar for a total of six weeks. He was followed up regularly in our clinic and showed clinical and radiological improvement, maintaining the alignment with no neurological deficits (Figure 5).

## Discussion

Anatomically, the vertebral artery has four segments: the first segment (V1) arises from the subclavian arteries, and the second segment (V2) ascends through the transverse foramina of the first six cervical vertebrae. As it exits the transverse foramen of C2, the vertebral artery turns laterally to pass through the transverse foramen of the atlas, where it subsequently curves posteriorly and medially over the posterior arch of the atlas and around the atlantooccipital joint, which is the third segment (V3). VA's fourth intracranial segment supplies PICA.

Carpenter, in 1961, was the first to describe the association between cervical spine fracture and VAI [5]. Although the V1 and V2 segments of the vertebral artery are surrounded and protected with osseous structures, it has the highest rate of VAI [5]. The suggested mechanism of VAI is direct trauma from bone fragments or excessive stretch in fracture dislocations, which can cause arterial injury. The prevalence of VAI following trauma varies considerably [3]. Formerly, VAI following blunt cervical trauma was thought to be relatively uncommon; however, improvements in imaging modalities have significantly increased rate of VAI diagnosis, such as digital subtraction angiography (DSA), magnetic resonance angiography (MRA), and computed tomography angiography (CTA). Because it is noninvasive to the vascular system and simple to use and has a high detection rate, CTA is a first-choice diagnostic tool for VAI. The diagnosis of VAI is challenging since the circle of Willis and the collateral blood flow of the contralateral VA and nondominant VA blockage rarely manifest any symptoms. Unlike dominant or bilateral VA, occlusion can cause fatal ischemic damage to the cerebellum and brainstem [2]. Geddes et al. recommended using the expanded Denver criteria to screen patients with upper cervical spine fractures [6].

A study related to the efficiency of the expanded Denver criteria in detecting blunt cerebrovascular injury (BCVI) was conducted by Beliaev et al. [7]. They concluded that the expanded Denver protocol could identify trauma patients at risk of BCVI with a sensitivity of 97% and a specificity of 42%. Another significant study finding is the negative predictive value of screening with the expanded Denver protocol, which excluded BCVI in trauma patients with a 99.8% probability [7]. The management of VAI in asymptomatic patients remains controversial since there is no class I and class II evidence to assist in the decision-making. Depending on the case, treatment options for VAI include anticoagulation, antiplatelet, endovascular intervention, surgical vessel sacrificing, or no treatment at all. There seems to be no consensus about the principles of treatment for VAI, except for individualization based on the specifics of the patient's VAI, associated injuries, and risk of bleeding [8]. In a study conducted by Cothren et al. on 235 patients diagnosed with VAI and blunt carotid injuries, they reported that antithrombotic therapy (anticoagulant and antiplatelet agents) had reduced the risk of ischemic brain insult from 21% to 0.05% [9].

## Conclusions

Our case is uncommon in that incidental finding of left PICA occurred in an asymptomatic patient after a Jefferson fracture. The clinician must have a high index of suspicion of vertebral artery injury in all upper cervical spine fractures. We recommend close observation and antithrombotic therapy for all VAIs. 
